# Providing Standardized Gene Nomenclature for Domestic Ungulate Scavenger Receptor Genes

**DOI:** 10.17912/micropub.biology.001584

**Published:** 2025-06-24

**Authors:** Jessica Cruz, Fiona McCarthy

**Affiliations:** 1 University of Arizona, Tucson, Arizona, United States; 2 Animal Comparative and Biomedical Sciences, University of Arizona

## Abstract

Scavenger Receptor (SCAR) genes are a heterogeneous group involved in innate immunity and immunomodulation. While standardized gene nomenclature is provided for model organism SCAR genes, ungulates have novel SCAR genes which have not been systematically classified. This study compares SCAR genes for five representative ungulate species which are selected based on their agricultural importance and representation. We identify and classify twenty-seven SCAR genes common to all species and provide standardized gene nomenclature for genes novel to these ungulate species. Notably, we provide standardized gene nomenclature for the previously studied Workshop Cluster 1 (WC1) genes which are absent in model organisms.

**Figure 1. Comparison of gene order for the SCARI (WC1) gene cluster across key vertebrate species.**
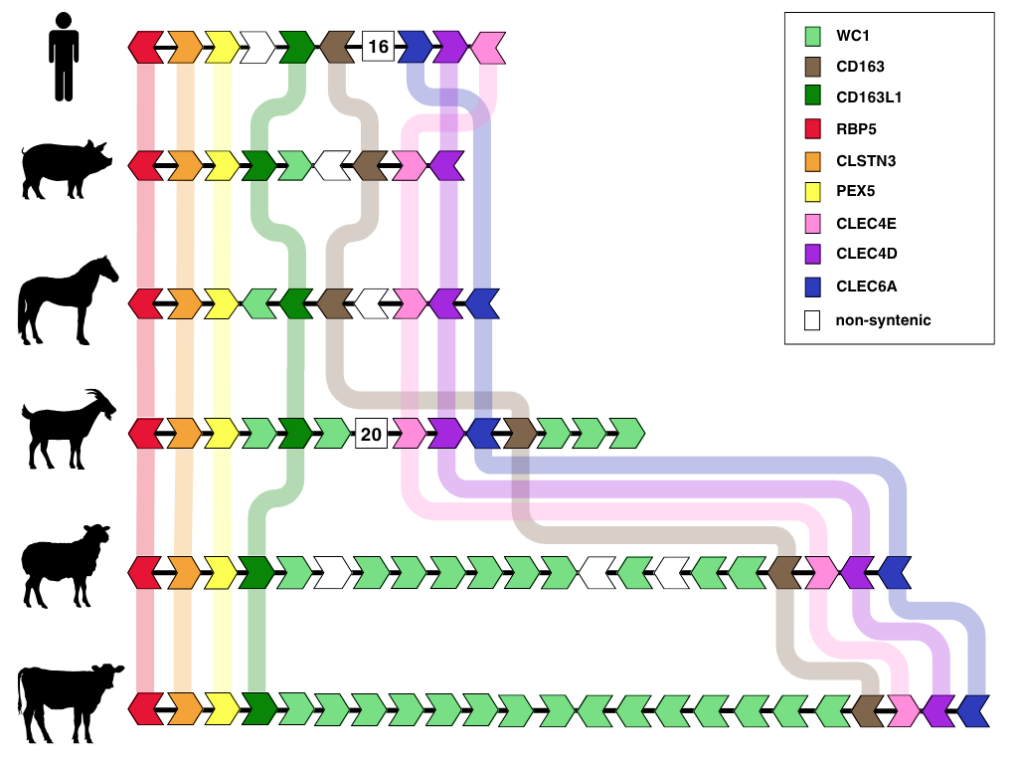
Species listed from top to bottom include *Homo sapien, Sus scrofa, Equus caballus, Capra hircus, Ovis aries,* and *Bos taurus* . Genes are shown as arrows representing orientation. Orthologous genes are shown in the same color, with corresponding shaded lines connecting them across species. Flanking genes (RBP5, CLSTN3 and PEX5 on the left, CLEC4E, CLEC4D and CLEC6A on the right) surrounding the cluster are included to demonstrate the synteny around this gene expansion. *Sus scrofa’s* gene order is based upon the Ensembl annotation, while in other species there was no difference in the gene annotation provided by Ensembl and NCBI. White arrows indicate genes that are unrelated to this study.

## Description

Scavenger Receptor (SCAR) genes are a heterogeneous group of cell-surface receptor genes involved in innate immunity and immunomodulation (Alquraini & Khoury, 2020). In humans, scavenger receptors are further classified into twelve subgroups based on structure and function (Alquraini & Khoury, 2020; PrabhuDas et al., 2017). However, SCAR genes are present across a wide range of animals, and nonhuman vertebrates have an expanded repertoire of some of these receptors (Herzig et al., 2010). This project provides standardized gene nomenclature for previously unassigned SCAR genes found across multiple ungulate species. We selected cattle, horses, pigs, sheep and goats as specific of agricultural importance whose SCAR genes have been previously studied. Our experimental approach is to identify the SCAR gene repertoire in these species and provide standardized gene nomenclature which reflects what is known about the function of these genes, particularly SCAR genes not found in humans. We used sequence similarity, motif analysis and phylogenetic analysis to identify SCAR genes in cattle, horses, pigs, sheep and goats. Gene synteny and phylogenetic analysis were used to determine relationships between mammalian SCAR genes while standardized gene nomenclature was assigned using VGNC guidelines (Jones et al., 2023). For SCAR genes not found in humans we reviewed publications to determine function and assess names which have previously been used by the research community. The proposed names for these novel genes better reflect the function of their gene products and their probable roles in innate immune responses.

We identified 27 genes orthologous to human SCAR genes. In addition, we identified a novel group of SCAR genes found in ungulate species, but absent in humans, that have previously been described in cattle as Workshop Cluster 1 (WC1) genes (Morrison & Davis, 1991). WC1 genes are conserved across multiple ungulate genomes but have different copy numbers (16 in cattle, 13 in sheep, 8 in goat, 9 in pig and 1 in horse). Sequence searches of vertebrate proteins demonstrated that WC1 proteins are found in all mammals except primates and rodents. These genes were previously determined to be structurally and functionally related to human CD163 and CD163L1 (Herzig et al., 2010). WC1 genes are expressed on gamma delta T cells as transmembrane co-stimulatory receptors involved in pathogen pattern recognition and inflammatory response initiation (Hedges et al., 2003; Wang et al., 2011). Figure 1 shows the conserved WC1 cluster in ungulates, depicting this gene expansion which is absent in the human genome. In addition to this cluster of WC1 genes in the ungulates, cattle, goat and pig have additional WC1 genes which are located on unplaced contigs, which can be placed within this WC1 cluster in their respective genomes.

While most of the species in this study show synteny with the human genes in this region, the downstream gene order in the current goat genome is not well conserved, with the WC1 cluster split by the conserved CLEC and CD163 genes. In addition, three goat WC1 genes are found on unplaced contigs within the goat genome ( *Capra hircus* ARS1.2). The unplaced sequences in the WC1 cluster indicate poor assembly in this region, which may also impact the observed lack of gene synteny, particularly in comparison to the gene synteny in the closely related sheep genome. Likewise, the gene synteny in pigs does not match the observed gene order when compared to other ungulates. Pig CD163 is annotated downstream of the single placed WC1 gene while CD163L1 is annotated upstream, and this section of the genome appears to be flipped in the NCBI annotation. Both the NCBI and Ensembl annotations of the pig genome do not include CLEC6A. While only one WC1 gene is placed in this region, we identified seven additional WC1 genes on unplaced contigs, and this is in agreement with previous studies (Le Page et al., 2018; Le Page et al., 2019). This serves as further evidence that this region has assembly errors in the pig genome ( *Sus scrofa* Sscrofa11.1). We note that the gene order in the Ensembl assembly (Sscrofa11.1) is accurate when compared to our observed genomes, as shown in Figure 1.

WC1 is expressed in a subpopulation of CD4 ^-^ CD8 ^-^ T cells called gamma delta T cells (Morrison & Davis, 1991). In humans, most T cells develop in the thymus and have a T cell receptor (TCR) composed of alpha beta chains (Fozza et al., 2017). However, a small population of gamma delta T cells localized to peripheral tissues (skin, mucosa, intestine) was identified in humans, differentiated in receptor structure via gamma and delta chains (Bank et al., 1986; Brenner et al., 1986; Hu et al., 2023). The gamma delta TCR can rapidly and directly interact with various pathogens without prior antigen exposure (Tanaka et al., 1995), exhibiting characteristics of both innate and adaptive immunity. Much like the alpha beta TCR, the gamma delta TCR signals through the CD3 complex, which binds to antigen-presenting non-classical MHC and related molecules (Luoma et al., 2013). However, the gamma delta TCR may also recognize pathogens directly, without the use of antigen-presenting molecules, and may initiate an immune response on its own (Chien & Konigshofer, 2007; Morath & Schamel, 2020). WC1 genes contribute to immunity as gamma delta T cell-specific co-receptors involved in varied pathogen recognition and cell activation accompanied by the CD3 complex (Hsu et al., 2015). Vertebrate species with an expanded repertoire of WC1 genes have a larger subpopulation of gamma delta T cells in the peripheral blood, ranging from 30-50% of T cells especially in young ruminants (Hein & Mackay, 1991). In primates and rodents which naturally lack WC1 genes, CD163L1 or SCART1 respectively act as functional equivalents to WC1 (Herzig et al., 2010).

Based upon scavenger receptor classification, we propose that WC1 genes be labelled as scavenger receptor class I member (SCARI). We propose gene nomenclature in line with the recommended protein nomenclature (PrabhuDas et al., 2017). We applied this numbering system, and numbered the additional ungulate genes according to gene synteny and phylogenetic inference. This system of standardized nomenclature agrees with VGNC guidelines (Jones et al., 2023) and feedback from community experts, and is extensible to genes in other species.

## Methods

Human SCAR genes were defined using the HGNC gene group set. We identified orthologous SCAR genes between human and representative domestic ungulates ( *Equus caballus* EquCab3.0, *Sus scrofa* Sscrofa11.1, *Bos taurus* ARS-UCD2.0, *Ovis aries* ARS-UI_Ramb_v3.0, and *Capra hircus* ARS1.2) using NCBI and Ensembl ortholog prediction resources. In cases with a direct 1:1 ortholog, standardized gene nomenclature was applied to the ungulate gene based upon the corresponding human gene nomenclature. Additional names used for these genes in the published literature were collected by manual review of the literature associated with each gene.

Additional ungulate SCAR genes with no human orthologs were identified by sequence similarity searches using human SCAR proteins to search the NCBI species-specific protein databases, followed by manual review to identify proteins with scavenger receptor motifs. Ungulate SCAR genes with no human ortholog were assigned standardized nomenclature based upon gene synteny, phylogenetic analysis, and review of associated literature. The gene order of WC1 genes were manually reviewed, using three conserved mammalian genes on either side of the gene cluster; CLEC6A, CLEC4D, CLEC4E and PEX5, CLSTN3, RBP5.

Nomenclature standardization from amongst ungulate species is based upon phylogenetic analysis and review of gene order. The longest protein sequence was selected as the representative protein sequence for phylogenetic analysis. Protein sequences were imported into Geneious Prime 2024.0.7 and aligned using Geneious Alignment (with free end gaps and matrix Blosum62). Phylogenetic trees were created from the alignment through the Geneious Tree Builder (Jukes-Cantor, Neighbor-Joining) and 100 bootstrap replicates. Bovine WC1 genes were numbered in line with established protein nomenclature (PrabhuDas et al., 2017), with numbers not identified in the current bovine annotation reserved for future use. Additional bovine WC1/SCARI genes were then numbered based upon their gene order and annotation. Following the assignment of gene order in bovine, phylogenetic analysis was performed to determine likely orthologs in the remaining ungulates. Strict orthologs were assigned standardized names, while the remaining genes were numbered consecutively according to gene order along equine chromosome 6, swine chromosome 5, ovine chromosome 3 and caprine chromosome 5. Genes found on unassigned sequences were named lastly in respective assemblies.

## Data Availability

Description: This dataset presents the orthologs of the SCAR and related WC1 genes across genome assemblies described within this study. Provided are the NCBI and Ensembl gene IDs, current gene nomenclature at the time of this study, our proposed nomenclature, and known aliases.. Resource Type: Dataset. DOI: https://doi.org/10.22002/fbz4s-mdw82
